# Supplementary material for: Piceatannol Prevents Obesity and Fat Accumulation Caused by Estrogen Deficiency in Female Mice by Promoting Lipolysis
Source: Nutrients. 2023 Mar 12;15(6):1374. doi: 10.3390/nu15061374 (PMC10056039; doi:10.3390/nu15061374)
Supplement: Supplementary file 1 [file nutrients-15-01374-s001.zip › Table S1.pdf]

Table S1. Composition of the experimental diets.

	High-Fat Diet (HFD)	0.25%PIC-HFD
Casein	25	25
L-cystine	0.375	0.375
Corn starch	10.17	9.92
α Corn starch	22	22
Sucrose	10	10
Soybean oil	2.5	2.5
Lard	20.2	20.2
Cellulose	5	5
AIN-93G Mineral mix	3.5	3.5
AIN-93G Vitamin mix	1	1
Choline bitartrate	0.25	0.25
tert-Butylhydroquinone	0.005	0.005
Piceatannol	-	0.25

(g/100 g diet)
