# Supplementary figures and images for: Piceatannol Prevents Obesity and Fat Accumulation Caused by Estrogen Deficiency in Female Mice by Promoting Lipolysis
Source: Nutrients. 2023 Mar 12;15(6):1374. doi: 10.3390/nu15061374 (PMC10056039; doi:10.3390/nu15061374)

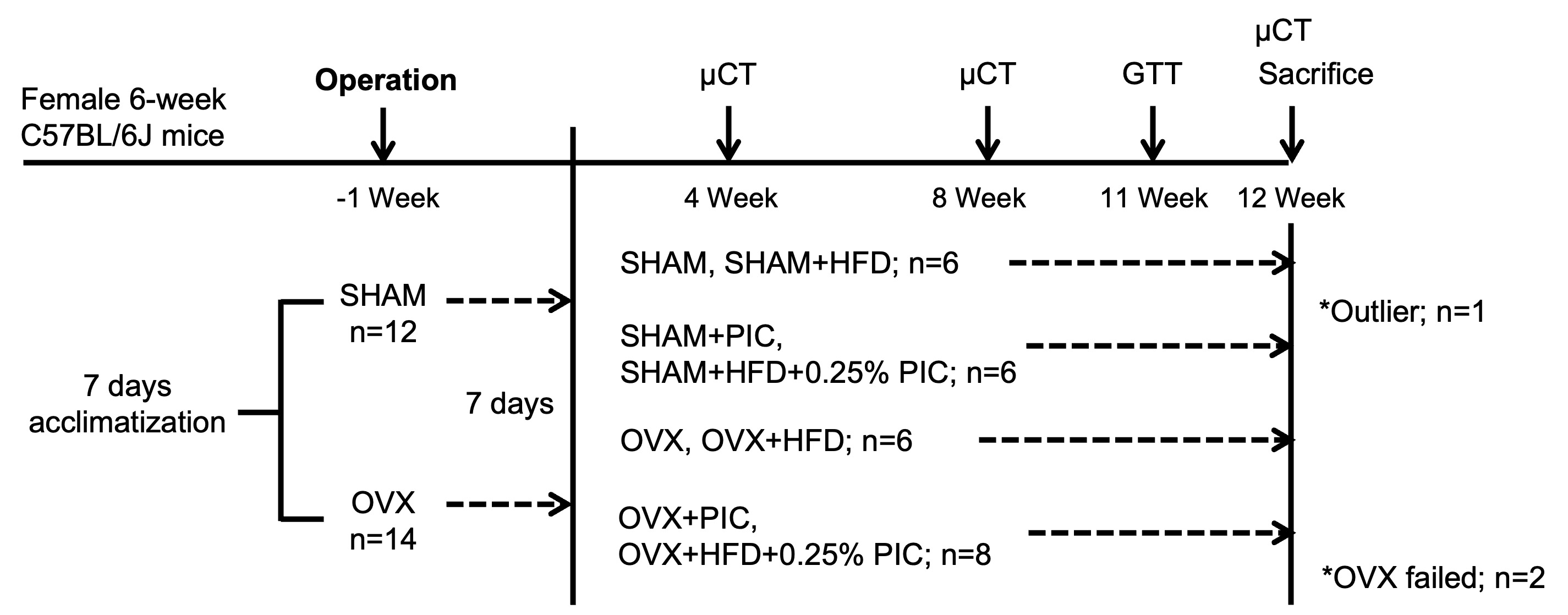

Supplement: Supplementary file 1 [file nutrients-15-01374-s001.zip › Figure S1.jpg]
